# Binocular rivalry reveals differential face processing in congenital prosopagnosia

**DOI:** 10.1038/s41598-024-55023-7

**Published:** 2024-03-20

**Authors:** Theresa Halder, Karin Ludwig, Thomas Schenk

**Affiliations:** Clinical Neuropsychology, Department of Psychology, Ludwig-Maximilians-Universität München, Leopoldstraße 13, 80802 München, Germany

**Keywords:** Psychology, Neuroscience

## Abstract

Congenital Prosopagnosia (CP) is an innate impairment in face perception with heterogeneous characteristics. It is still unclear if and to what degree holistic processing of faces is disrupted in CP. Such disruption would be expected to lead to a focus on local features of the face. In this study, we used binocular rivalry (BR) to implicitly measure face perception in conditions that favour holistic or local processing. The underlying assumption is that if stimulus saliency affects the perceptual dominance of a given stimulus in BR, one can deduce how salient a stimulus is for a given group (here: participants with and without CP) based on the measured perceptual dominance. A further open question is whether the deficit in face processing in CP extends to the processing of the facial display of emotions. In experiment 1, we compared predominance of upright and inverted faces displaying different emotions (fearful, happy, neutral) vs. houses between participants with CP (N = 21) and with normal face perception (N = 21). The results suggest that CP observers process emotions in faces automatically but rely more on local features than controls. The inversion of faces, which is supposed to disturb holistic processing, affected controls in a more pronounced way than participants with CP. In experiment 2, we introduced the Thatcher effect in BR by inverting the eye and mouth regions of the presented faces in the hope of further increasing the effect of face inversion. However, our expectations were not borne out by the results. Critically, both experiments showed that inversion effects were more pronounced in controls than in CP, suggesting that holistic face processing is less relevant in CP. We find BR to be a useful implicit test for assessing visual processing specificities in neurological participants.

## Introduction

Prosopagnosia can be the consequence of brain lesions (acquired prosopagnosia) but can also exist from birth without any detectable brain damage. In the latter case, it is called congenital prosopagnosia. Congenital prosopagnosia (CP) is characterized by a selective face perception impairment while other cognitive abilities are intact^1–5^. The terms ‘congenital’ and ‘developmental’ prosopagnosia are sometimes used synonymously by experts. Here we will use the term ‘congenital prosopagnosia’ (CP). Estimated prevalence rates of this congenital form range from 0.64 to 5.42%^6–8^. Reports of familial accumulations indicate that, in some cases, a genetic component might play a role^9,10^. The severity of CP varies widely between those affected, from mild to severe impairment^11,12^. In severe cases, even faces of close family members cannot be recognized^3^. Often, cues such as hairstyle, posture, or voice are used to compensate for the impairment and to reduce recognition failures in daily life^13^. Despite many years of research to determine and define how exactly CP face processing differs from the normal case, empirical results do not provide a clear answer^1,14^. This is partly because to date the process of normal face perception is not fully understood.

### Measuring holistic face processing using inversion and the Thatcher effect

A predominant theory of face perception is the theory of ‘holistic face processing’. It states that faces are generally processed globally, meaning that the *relation between* local features rather than the features themselves are used to identify faces. A consequence of this strategy is that local features are somewhat neglected^15–17^. The observation of the face inversion effect supports this theory^18–20^: when participants with normal face perception are asked whether two faces are identical, they make more errors when the faces are presented upside down. Importantly, this error is larger for inverted faces than for inverted objects (therefore an indirect measure of holistic processing). Inversion is unlikely to affect the efficiency with which local facial features and their configuration can be assessed. In short, inversion seems to be a manipulation that selectively impairs holistic processing. Thus, the fact that observers struggle to match faces in an inverted position suggests that neurotypical observers make heavy use of the holistic appearance of faces. One account of CP assumes that this holistic process is selectively impaired^3,21,22^. If true, we might expect that face-inversion affects performance in CP observers less than in observers with normal face perception. This prediction was confirmed in several studies where inversion had less impact on the performance of CP observers^21,23,24^ although there are studies that report a substantial variability in performance of CP observers in face-inversion tasks^25,26^.

A specific inability to use holistic information could also explain why CP observers are less affected by the *Thatcher* effect. In the Thatcher effect (named after the first female British prime minister, Margaret Thatcher, whose face was used for the first demonstration of this effect), the eye and mouth regions are rotated by 180° within the face. Neurotypical observers immediately see the obvious and grotesque change in appearance when the face is presented in the canonical, upright position (see example in Fig. 2). However, they fail to do so when the face is presented upside down, confirming the orientation-specific nature of holistic processing^27,28^. There are reports that people with CP are less susceptible to the Thatcher-effect but also less susceptible to the inversion effect and can reliably detect the aberrant arrangement even in the inverted version^29,30^.

However, other studies challenge the explanation of disturbed holistic processing in CP by showing that some aspects of holistic face perception in CP are indistinguishable from those of controls. One example is provided by the composite face effect, which is a direct measure of holistic face processing. In the composite face effect, top and bottom halves of different facial identities are aligned to form one face, which is then perceived as a novel facial configuration. This illusion provides a powerful demonstration of the holistic nature of face processing: when two composite faces are presented with the same top half in both composites, observers find it difficult to see that the top halves are indeed identical^31^. It seems that a change in the bottom half changes how the top half is perceived, confirming that faces are processed as a whole, not as a sum of its parts (however, it should be noted that the effect and its interpretation are not beyond criticism; for a review, see Ref.^32^). Contrary to what was found with other holistic-face-processing effects, Biotti and colleagues^33^ showed that the composite face effect had a similar magnitude in controls and in participants with CP (N = 16). A predominantly normal composite face effect was also found in a smaller cohort reported by Le Grand and colleagues^34^. A further study used the part-whole-task^35^ in which feature changes (i.e., change of the nose) had to be detected when presented as part of a whole face or in isolation^36^, a task in which participants usually perform better in the whole-face condition. They found that participants with a mild impairment in face processing can profit from holistic information just as controls do.

### Perception of emotional faces

Another matter of debate is whether people with CP also display an impairment in facial emotion perception. One might suspect that observers who struggle even with basic face processing tasks, such as recognizing the identity of face, might struggle even more when given the task to recognize the emotion expressed by a face. Furthermore, it was hypothesized that facial emotion recognition depends on holistic information, i.e., the very process that is assumed to be deficient in CP^37–39^. Indeed, there are reports of significant deficits in emotion recognition in CP^2,10,40–48^. It is therefore surprising that several studies reported unimpaired emotion recognition in CP, suggesting that emotion recognition and face identity recognition are distinct processes^10,49–51^.

### Performance measures

Most of the studies mentioned have used performance measures to assess CP. The problem with these measures is that the aim is obvious to the participants, and they will thus attempt to perform as well as possible using whatever strategy proves most efficient. Participants with CP are known to develop and use strategies to mitigate the consequences of their impairment^50,52–54^. They might use voice, hairstyle, individual face forms, features, or blemishes to identify a person. Most of these strategies are not available in the experimental situation since the faces are shown in isolation and without identifying marks such as moles. The participants might, however, still rely heavily on the individual facial features and the form of the face. A normally sighted and a colour-blind observer might recognize the signal in a traffic light with comparable accuracy and speed but will likely use different perceptual cues and processes (i.e., colour in the case of the normally sighted person, and light-position in the colour-blind person). The same might be true for CP: affected and unaffected observers might do equally well in face-perception tasks but achieve this by different means. This is the downside of explicit perceptual measures. A good strategy to uncover some of the processing differences between CP and unaffected observers might thus be to use measures that focus on the process rather than the outcome of a perceptual task, assess physiological variables or use measures that relate to behavioral aspects that are either not consciously controlled or not part of the instructed task^55,56^. We will call such measures implicit measures. Tranel and Damasio^57^ used skin-conductance measures to reveal preserved yet consciously inaccessible face-familiarity processing in participants with acquired prosopagnosia. Others employed event-related potentials (ERP) to find evidence of subconscious face-processing in the same group^42,58,59^ or employed eyetracking methods^60,61^. Overall, however, the use of explicit performance measures in research on CP is still prevalent. We wish to introduce another implicit measure for the study of face processing, namely binocular rivalry (BR).

### Binocular rivalry

BR occurs when the two eyes are presented with two different images. Conscious perception then alternates between these two images or—less often—consists of a mixture of both images^62–64^. It has been shown that the relative stimulus strength determines which stimulus is consciously perceived for a longer period of the presentation time^63^. For example, high contrast images gain advantage over low contrast stimuli^65^ and moving patterns predominate over still images^66^. Apart from low level image properties, predominance is also influenced by object-related, configural properties of the stimulus. Meaningful patterns lead to longer dominance intervals than random patterns but lose their predominance when inverted^67^. Face stimuli have been used in several studies to investigate preferential processing of faces. In BR, face images show strong dominance when compared to meaningless random dot patterns but also meaningful objects like houses^67,68^. Moreover, there is evidence of sensitivity for the socially significant information in faces in BR. Processing under BR is modulated by emotional expressions (happy and fearful^68^, surprised^69^, and disgusted^70^), with emotional facial expressions being dominant for longer periods than neutral ones. These studies showed that the face inversion effect is also measurable in BR. When presenting the rivalling face-house pairs, upright fearful and happy faces were significantly more dominant than neutral faces, but with inverted stimulus presentation, the impact of facial emotion was lost^68^. The faces shown upright and inverted differed only in this respect and were otherwise identical. Thus, bottom-up saliency was identical. Altogether we can assume that saliency and thus the predominance of visual input in BR is also affected by higher level image properties such as meaning, familiarity, information value, and social aspects^71^. Using BR to study CP participants, we can implicitly measure how the saliency of faces might be different in CP and how perception is affected by the ready availability of global and local features.

To sum up, we wish to use BR as a measure of how salient a given face (upright/inverted, neutral/emotional, normal/thatcherized) is for a given group (controls/participants with CP). Thus, binocular rivalry might help us to uncover the extent to which CP observers use global versus local features in face perception and how this affects their decoding of facial emotions.

### Goals and hypotheses of this study

Our primary aim was thus to explore the potential of BR as an implicit method for uncovering differences in perceptual processing styles between controls and CP observers. As described above, a key advantage of an implicit approach is that it can circumvent the influence of compensatory strategies that are assumed to play a substantial role in the performance of people suffering from CP. Specifically, we wished to address three questions.

First, we tested whether CP observers rely less on holistic processing strategies than controls in general. If true, one might expect that the inversion effect on face predominance during BR is less pronounced in CP participants than in controls. This hypothesis has been addressed in previous studies. Earlier studies found mixed results. We hope that by using an implicit measure we might obtain findings that are less affected by the use of compensatory strategies and thus more consistent. In general, we would argue that it is important to examine a given research hypothesis in multiple studies with a variety of different research techniques to enhance confidence in the theoretical conclusions and provide opportunities to uncover novel aspects.

Secondly, we wished to explore if the decoding of emotional expressions in faces is indeed preserved in CP observers, as some studies suggest. We hypothesized that CP observers also show a modulation of perceptual predominance by facial emotion but rely more on local features and less on holistic aspects in their decoding of emotions in faces than controls. Thus, for upright faces we expect a modulation of face predominance in BR by the emotion expressed in those faces for both groups. We also expect that this modulation will be substantially dampened in controls in the inverted condition and that this dampening in the inverted condition will be less pronounced in CP observers. Finally, we wanted to explore the possibility that the thatcherized faces might render the BR paradigm even more sensitive to uncover differences in perceptual strategies for controls versus CP observers. The rationale behind this is the following: (1) In neurotypical observers the inversion effect is predicted to be particularly striking when thatcherized faces are used: its aberrant nature might render thatcherized faces even more salient and dominant than normal faces. Furthermore, inversion almost completely destroys the effect of thatcherization in neurotypical observers. Thus, one might expect a particularly strong inversion effect, when BR dominance is measured in neurotypical observers. (2) CP observers in contrast are much less affected by the Thatcher effect to begin with and also detect the Thatcher-manipulation with comparable ease in both upright and inverted conditions. Taken together, we expect a particularly striking difference in the effect of inversion on BR performance between neurotypical and CP observers when thatcherized faces are used.

## Materials and methods

### Participants

Twenty-one participants with congenital prosopagnosia (CP: 6 male, 15 female; mean age 42.6, SD = 15.9) were selected according to the procedure described below. The experimental data of one further CP participant could not be analyzed due to technical issues during the experiment. In addition, twenty-one participants with normal face recognition abilities were recruited for the control group (CG: 6 male, 15 female; mean age 48.1, SD = 11.6). One potential further control participant was excluded after the first minutes of the experiment because of strabismus which made participation in the BR experiment unfeasible. Groups did not differ significantly regarding age [t(40) = − 1.27, p = 0.21] or gender [chi^2^(1) = 0.12, p = 0.73]. Table 1 contains demographic data of both groups. The test protocol was approved by the ethics committee of the psychology department of the Ludwig-Maximilians-Universität München. All methods were performed in accordance with the given guidelines and regulations. All participants had normal or corrected-to-normal vision and gave written informed consent prior to testing including consent for publication of data in an online open-access publication. Participants with any history of brain injury or neurodegenerative disease were excluded. Additionally, we did not include participants with diagnosed autism spectrum disorder, as autism may correlate with changes in facial emotion perception^72^.

**Table 1:** Test results of all participants.

Group	No	Age, sex	PI-20	CFMT %	Benton	MTF	Sum score	Ekman
CP	1	47, m	**72**	**40**	**32**	**21**	4	53
	2	47, m	**67**	**54**	**34**	23	3	53
	3	50, m	**81**	65	**38**	22	2	46
	4	50, m	**74**	61	**35**	23	2	49
	5	66, m	**73**	**49**	**34**	23	3	37
	6	31, m	**70**	**47**	**25**	**21**	4	50
	7	29, f	**79**	**47**	**25**	22	3	47
	8	33, f	**91**	79	**33**	23	2	51
	9	35, f	**74**	83	**38**	24	2	54
	10	38, f	**70**	**58**	39	**20**	3	40
	11	39, f	**71**	83	**27**	24	2	52
	12	46, f	**92**	61	**33**	24	2	51
	13	47, f	**80**	**47**	**30**	**21**	4	47
	14	47, f	**94**	63	**21**	24	3	50
	15	47, f	**68**	69	**32**	25	2	48
	16	47, f	**75**	**48**	**33**	25	3	49
	17	52, f	**72**	**57**	**23**	24	3	47
	18	53, f	**68**	**58**	39	**21**	3	57
	19	57, f	**90**	**46**	**26**	**19**	4	46
	20	60, f	**73**	**51**	**29**	25	3	53
	21	69, f	**69**	61	**30**	25	2	45
		**Mean (SD)**	**76 (8.6)**	**58 (12.2)**	**31 (5.3)**	**22 (1.8)**		**48 (4.6)**
CG	22	47, m	23	**46**	41	25	1	52
	23	70, m	34	**59**	44	25	0	51
	24	34, m	36	**59**	44	25	0	54
	25	62, m	48	65	51	25	0	53
	26	35, m	25	**59**	50	25	0	55
	27	25, m	24	70	51	25	0	51
	28	21, f	29	65	44	25	0	55
	29	23, f	25	**55**	52	25	1	55
	30	32, f	34	83	47	25	0	55
	31	38, f	35	71	46	24	0	42
	32	39, f	23	90	44	25	0	56
	33	60, f	34	**55**	41	25	1	55
	34	61, f	30	90	49	25	0	49
	35	50, f	45	**51**	41	25	1	59
	36	53, f	27	70	51	25	0	56
	37	19, f	29	79	41	25	0	44
	38	25, f	36	**55**	49	25	1	52
	39	37, f	33	91	41	25	0	47
	40	65, f	24	65	50	25	0	47
	41	35, f	41	95	43	25	0	51
	42	68, f	38	70	50	24	0	53
		**Mean (SD)**	**32 (7.2)**	**69 (14.4)**	**46 (4)**	**24 (0.3)**		**52 (4.2)**

Values outside the normal range are in bold.

*CP* congenital prosopagnosia, *CG* control group, all participants had a score of 98 in the Corvist: Cortical Vision Screening Test. PI-20: Prosopagnosia Index 20, *CFMT* Cambridge Face Memory Test, *Benton* Benton Facial Recognition Test, *MTF* Short Recognition Memory Test for Faces from the Camden Memory Test, Ekman: Ekman 60 Faces Test (FEEST), *m* male, *f* female. The *Sum Score* indicates the number of test-results outside the cut-off values of the five perceptual tests (PI-20, CFMT, Benton, MFT). The Ekman test results are not included in the Sum Score.

### Neuropsychological tests

CP Participants were largely recruited through an interview on the topic of face recognition and prosopagnosia given by one of the authors on a local radio station at the end of which people were invited to fill out our online questionnaire (i.e., the German version of the twenty-item CP index (PI-20), original version by Shah and colleagues^73^). The PI-20 is a subjective questionnaire containing 20 items that explicitly and implicitly queries the personal impression of face perception, recognition, and memory with high validity^74,75^. Based on correlations between this self-report and objective tests of face perception, PI-20 scores of 65 and higher are indicative of prosopagnostic traits (2SD above mean of controls).

Thus, people with a value above 65 were invited to our laboratory for further testing. With one exception (see above) all invited participants were included in our study. Said participants were tested with three additional face perception tests and included in the CP group if one or more test scores including the PI-20 indicated an impairment in face perception. We chose to adopt these more liberal inclusion criteria in response to recent discussions in publications that suggest self-reported prosopagnosia as inclusion criteria^11,75^. In the following we provide a detailed description of the employed tests. It is interesting to note that all participants with above-cut-off values in the screening questionnaire (i.e. above 65) also had clear deficits in at least one of the employed lab-based face-perception tests. This striking agreement between the subjective ratings and objective test results can possibly be attributed to the fact that all participants had previously listened to the radio interview detailing the typical diagnostic signs of CP.

All 42 participants completed a neuropsychological examination. Basic visual, facial, and emotional perception was examined with established neuropsychological tests specified below. Table 1 includes results of all tests. The participants with CP were selected by the following procedure.

Participants completed an online, German version of the twenty-item CP index (PI-20) (original version by Shah and colleagues^73^). The PI-20 is a subjective questionnaire containing 20 items that explicitly and implicitly queries the personal impression of face perception, recognition, and memory with high validity^74,75^. For our study, participants with a score above 65 points in the PI-20 were tested with three additional face perception tests and included in the CP group if two or more test scores exposed face perception impairment. In the following we provide a detailed description of the employed tests.

The Cortical Visual Screening Test (CORVIST)^76^ screens for impairments in early visual processing such as perception of colours or shapes. Any errors would require closer examination of the respective ability.

The Cambridge Face Memory Test (CFMT) is a widely used computer based and time-restricted test where participants need to memorize 6 male target faces and identify these faces in sets of distractor faces^11,77,78^. Task difficulty is increased by rotating and blurring the test faces. According to the test developers, accuracy values below 58.47% are indicative of CP (2SD below mean of controls).

In the Benton Facial Recognition Test^79^ the observer must identify a target face out of a set of photographs of six faces without any time restriction. Scores are age corrected. As suggested by the test authors, participants with scores of 38 or lower (≤ 70.37%) are considered as moderately impaired, scores lower than 37 reflect severe impairment^79^.

In the Short Recognition Memory Test for Faces (MTF) from the Camden Memory Test^80^ the participant must estimate the age of 25 males and in the next stage, recognize these target faces when presented with a second but unfamiliar face. It should be mentioned that the MFT is not exclusively a test for facial memory as recognition is supported by cues beyond facial features (hair, cloths). A result below 22 (mean of normative data controls) was the cut-off.

To examine recognition of emotional facial expressions, all participants completed the Ekman 60 Faces test^81^. The computer-based test presents photographs of 10 actors showing the 6 different facial expressions (anger, disgust, fear, happiness, sadness, and surprise) in random order. The photographs are presented for 5 s each. After a photograph has disappeared, the participant selects the corresponding emotion.

Participants of the control group had to have a score below 65 in the PI-20 and be within the normal range in at least two of the three additional face perception tests. If a test exposed face perception impairment it counted as 1 point to the sum score. Control group participants were included with a sum score of 1 or less, CP participants with a sum score of ≥ 2 (see Table 1).

### Binocular rivalry

Binocular rivalry was induced by using commercial red-green stereo-glasses (anaglyphic glasses by American Paper Optics, recommended by Woods and Harris^82^ while presenting the rivalling stimuli in red and green. The two binocular rivalry experiments were programmed in Matlab (MathWorks, Natick, MA, Ref.^83^) using the Psychophysics Toolbox Version 3^84^. Stimuli were displayed in the middle of a 15.6-inch laptop monitor. They measured 2.5 × 3.6 cm (3.0° × 4.3°). Participants viewed the screen from a 48cm distance, controlled by a head rest. A patterned frame of 0.6° width was drawn around the stimuli to aid fusion. Low level image properties were matched for all stimuli pictures using the SHINE toolbox^85^. We matched the luminance of the red and green version of each stimulus with a photometer (Mavolux 5032C by Gossen). Rivalling stimulus pairs (house and face) were selected in a preliminary study to yield a minimum of mixed percepts or piecemeal rivalry. All participants completed both experiments in a dark and quiet environment.

### Experiment 1

**Figure 1:**
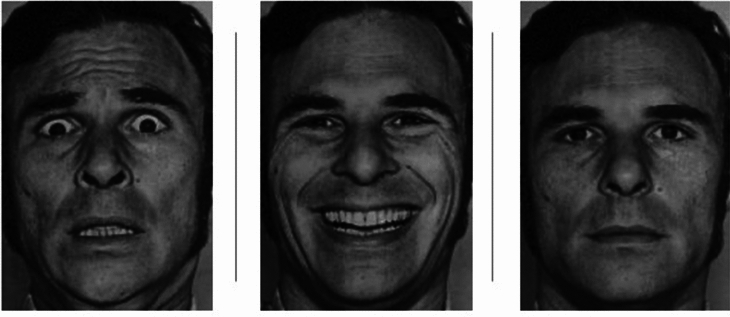
Example of face stimulus material used in Exp. 1 (fearful, happy, neutral) from the Ekman 60 faces test^71^. See supplement for complete stimulus set.

In the first experiment, participants were exposed to rivalling face-house pairs. We used pictures of four different persons (two females and two males) displaying three different emotions (happy, fearful, and neutral) from the Ekman 60 faces test^86^ (see Fig. 1 for examples of faces used. The whole set of stimuli can be found in the Supplementary Information). In practice trials, participants had the opportunity to familiarize themselves with the required perception-response assignment. During practice trials (and only there), unambiguous house and face stimuli and overlapping images of both (simulating mixed percept) were presented. This allowed us to confirm that all participants answered as intended. Half of the participants were asked to press the left arrow key as long as they perceived the house (and the right arrow key for the face), for the other half of participants the perception-response assignment was reversed. Participants were instructed to press no key when they had a mixed percept (i.e., a mixture of face and house features). Practice trials were not included in the analysis. Each trial lasted 60 s during which the participant had to continuously report their individual percept by pressing the key corresponding to their perceptual experience and by releasing the keys during times of mixed percepts. Participants had the option to take breaks between trials. The experiment consisted of four blocks, the order of which was completely randomized. Each block consisted of six different trials which resulted from the combination of three different facial expressions either presented upright or inverted. In each block the same face, the same house, and the same eye-filter-assignment (e.g., red filter in front of the right eye, green filter in front of the left eye) were used and the same stimulus-colour-assignment was employed (e.g., face presented in red, house in green). In each block, a new face and a new house and a different combination of the eye-filter- and the stimulus-colour-assignment were used (2 × 2). Thus, we ensured that each condition of interest (e.g., happy face, inverted) was shown in every possible stimulus-configuration so that we would average over possible confounds (like individual colour preferences or the influence of the dominant eye). Over participants, we varied the pairing of the face-house pair with the eye-filter- and stimulus-colour-assignment.

### Experiment 2

**Figure 2:**
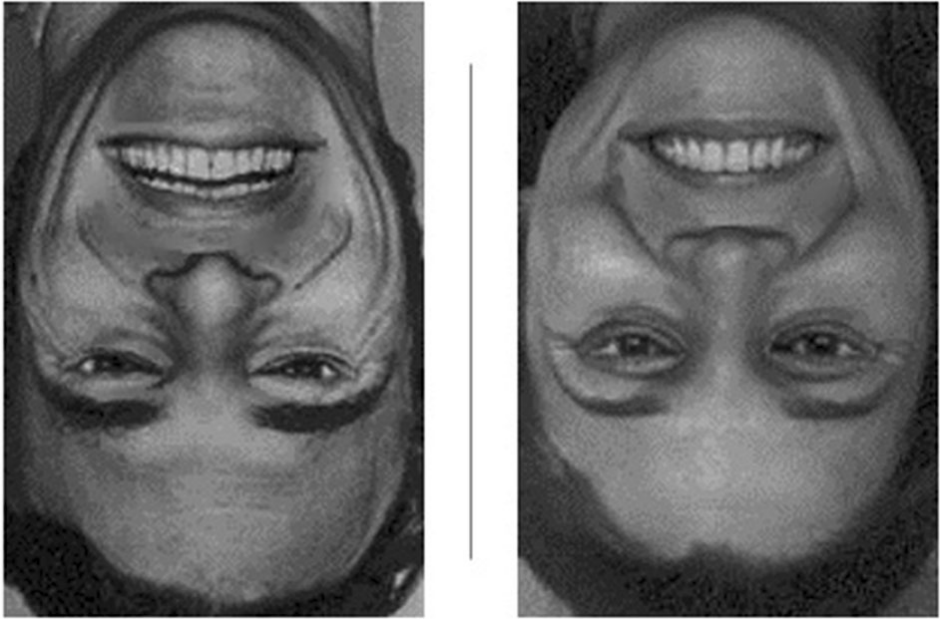
Example of thatcherized face stimulus material used in Exp. 2. Eye and mouth area are inverted in upside-down faces. See supplement for complete stimulus set.

In the second binocular rivalry experiment, we also used stimulus pairs of houses and faces but now the faces were presented either with the normal configuration of eyes and mouth (happy facial expression) or manipulated by rotating eye and mouth regions by 180° (thatcherized faces, see Fig. 2). The happy facial expression had to be used as the control, because the rotation of the smiling mouth results in the most striking Thatcher effect and of course, the control images had to be identical to the thatcherized images (other than the rotation of the eye and mouth region). We used one female and one male face. As in experiment 1, each trial lasted 60 s and the participants reported their percept using the same percept-key assignment as in experiment 1. The course of experiment 2 was almost identical to the one described for experiment 1 with a few exceptions. In experiment 2, each block consisted of eight trials (2 × 2 × 2: upright/inverted, thatcherized/not thatcherized, face red/green (house v.v.)). The eye-filter-assignment was repeated twice, resulting in four blocks.

### Data analysis

Data processing and analysis were performed using the commercial software packages MATLAB R2015a^83^ and SPSS (IBM SPSS Statistics for Windows, Version 25.0. Armonk, NY: IBM Corp.). Data visualisation was performed using Makie.jl^87^.

In a first step, total dominance durations for house and face percepts were summed separately for each participant in each trial and then averaged across trials. To calculate face predominance over all participants of each group, the facial dominance ratios were calculated for the three different emotions (experiment 1) or face modifications (normal vs. thatcherized, experiment 2) and presented separately for the upright and inverted orientation. Face predominance is defined as the ratio between the time during which the face dominated the percept divided by the time during which either the face or the house dominated (*face predominance* = T(total dominance face)/ [T(total dominance face) + T(total dominance house)]). The intervals during which no clear percept emerged (mixed percept) were excluded from the calculation.

## Results

### Neuropsychological characterisation of the participants

Unsurprisingly, given the selection criteria described in the methods section, all participants of the CP group described face recognition difficulties. Consistently, participants of the CP group had significantly higher results in their subjective evaluation of face impairment tested by the PI-20 than participants in the control group (CG: mean 32, SD 7.2; CP: mean 76, SD 8.6; t(40) = − 18.18, p < 0.001). The CP group had significantly lower scores in all three neuropsychological face perception tests: CFMT (CG: mean 69%, SD 14.4, CP: mean 58%, SD 12.2; t(40) = 2.5, p = 0.017), Benton (CG: mean 46, SD 4, CP: mean 31, SD 5.3; t(40) = 10.31, p < 0.001), and MTF (CG: mean 25, SD 0.3, CP: mean 23, SD 1.8; t(40) = 5.32, p < 0.001).

In the overall mean of the Ekman 60 faces test, participant 5 and 10 (CP) and 31 (CG), had overall scores slightly below the age-dependent cut-off, however these were not due to scores below the cut-off for the emotions “fear” or “happy” that were used in the experimental BR stimulus sets. The two groups differed in the overall Ekman score (CG: mean 52, SD 4.2, CP: mean 49, SD 4.6; t(40) = 2.3, p = 0.025). All participants, irrespective of group, achieved full CORVIST scores.

### Experiment 1

**Figure 3:**
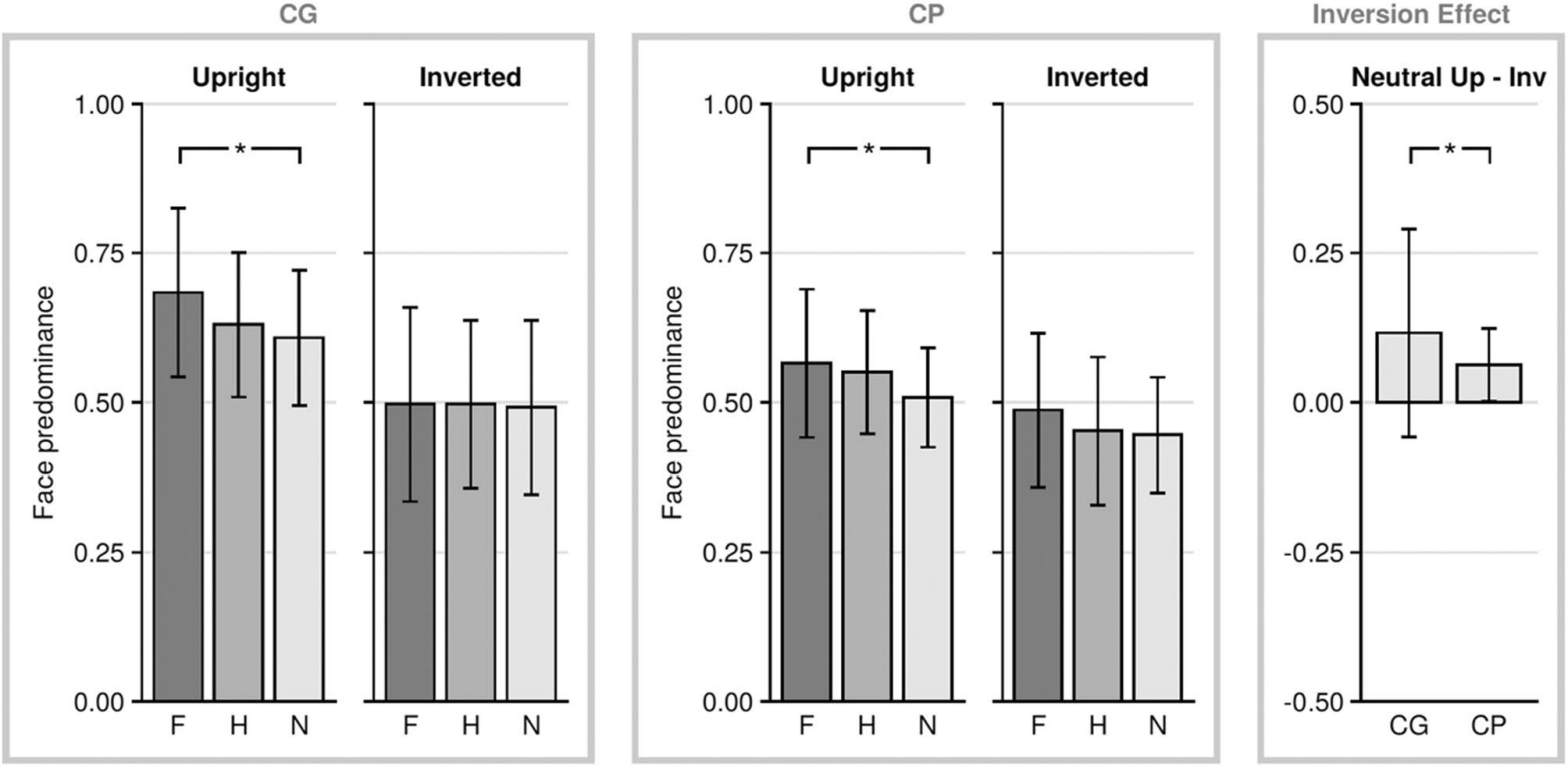
Face predominance of the two groups (*CG* control group, *CP* congenital prosopagnosia) for *F* fearful, *H* happy, and *N* neutral faces in both inversions (upright and inverted). *p < 0.05. The inversion effect (face predominance upright neutral faces—face predominance inverted neutral faces) was significantly smaller in CP. *p < 0.05.

We expected that CP observers rely more on local features of faces. Since local features are less affected by face-inversion than global configuration, we predicted face predominance to be less affected by the inversion in CP observers than in controls. Figure 3 shows the face predominance ratios of the two groups in all six conditions (see Table 2 for face predominance values of experiment 1). Inspection of the mean face predominance ratios in upright and inverted conditions shows several interesting trends: First, overall face predominance seems lower for CP than for controls. Second, the effect of inversion on both face-predominance and the modulation of face-predominance by emotional saliency seems more pronounced for controls than for CP observers. And third, emotion affects the predominance pattern for both control and CP observers.

**Table 2:** Face predominance values of experiment 1.

Emotion	Inversion	CG (N = 21) Mean *face predominance*	SD	CP (N = 21) Mean *face predominance*	SD
Fearful	Upright	0.684	0.141	0.565	0.124
	Inverted	0.497	0.162	0.487	0.123
Happy	Upright	0.630	0.121	0.550	0.103
	Inverted	0.497	0.140	0.452	0.123
Neutral	Upright	0.608	0.114	0.508	0.083
	Inverted	0.492	0.145	0.446	0.096

We computed a 3-way ANOVA with the factors group (CP, CG), emotion (fearful, happy, neutral) and inversion (upright, inverted) to examine which of the above trends are statistically reliable. The three-way-interaction was not significant (F(2,39) = 1.359, p = 0.269). We observed a tendency towards an interaction between inversion and group (F(1,40) = 3.765, p = 0.059, ηp^2^ = 0.086). This tendency—taken together with Fig. 3—suggests that the inversion effect was more pronounced for controls than for CP participants. The remaining interactions (inversion x emotion and emotion x group) were clearly non-significant (p > 0.1).

Regarding overall group differences, a significant main effect of group was found (F(1,40) = 5.254, p = 0.027, ηp^2^ = 0.116) which, together with Fig. 3, supports our fundamental hypothesis that face predominance in CP is lower than in controls. The effect of emotion was also significant (F(2,39) = 6.35, p = 0.004, ηp^2^ = 0.246) with higher face predominance for upright fearful as compared to upright neutral faces for both controls (0.684 ± 0.031 and 0.608 ± 0.025, respectively, p = 0.021, Bonferroni corrected) and CP participants (0.565 ± 0.124 and 0.508 ± 0.083, respectively, p = 0.018, Bonferroni corrected), as revealed by post-hoc tests. The effect of inversion was also significant (F(2,39) = 44.26, p < 0.001, ηp^2^ = 0.525), as the inversion of the stimuli led to a substantially smaller face predominance in both groups.

To investigate the effect of inversion separately from the factor emotion, we calculated the inversion effect for neutral faces (inversion effect = face predominance upright neutral faces—face predominance inverted neutral faces) for both groups (see Fig. 3). There was a significant difference between the inversion effect of CP and CG (t(40) = 1.324, p = 0.004, *d* = 0.41) showing that—compared to the control group—neutral face predominance was less affected by inversion in the CP group.

Bannerman and colleagues found in 27 participants that emotional saliency of faces (modulated by facial expression) had an impact on face dominance in the upright but not the inverted condition^68^. To see whether the same trend could be shown in our CG, we carried out a 2 × 3 ANOVA with the factor inversion (upright, inverted) and emotion (fearful, happy, neutral) on the control group data. We found a main effect of inversion (F(1,20) = 23.781, p < 0.001, ηp^2^ = 0.543) and a trend towards a significant interaction between the factors inversion and emotion (F(2,19) = 3.364, p = 0.056, ηp^2^ = 0.262). To follow up on this finding, we carried out two separate ANOVAs (each with the factor facial emotion) (1) with data from the upright and 2) with data from the inverted condition. There was a significant modulatory effect of facial emotion in upright faces (F(2,19) = 5.429, p = 0.008, ηp^2^ = 0.214) but not in case of the inverted faces (F(2,19) = 0.038, p = 0.963, ηp^2^ = 0.214). In sum, for the control observers we obtained a pattern of results that mimicked quite closely those reported by Bannerman and colleagues^55^.

We repeated the above analysis also for CP observers. We expected that the impact of inversion on face-predominance and its impact on the emotional-saliency effect will be reduced for CP observers. Inverting the faces also significantly reduced the mean face predominance in CP (F(1,20) = 24.82, p < 0.001, ηp^2^ = 0.554, Huynh–Feldt corrected), but the effect size of the CP inversion effect was smaller than in the CG. In the CP group, the interaction effect of the factors emotion and orientation was clearly not significant (F(2,19) = 0.560, p = 0.581); hence no follow-up analyses were conducted. Face predominance in CP was also affected by emotion, as shown by a significant main effect of emotion (F(2,19) = 4.722, p = 0.021, ηp^2^ = 0.191).

This shows that—although explicit emotion decoding (such as in a task requiring differentiating between emotions) is not necessary in BR—the results were nevertheless modulated by facial expression of emotion in CP. However, the non-significant interaction effect of the factors emotion and orientation suggests that this was based on processes that work in both upright and inverted faces, i.e., emotion extraction from local features.

### Experiment 2

Thatcherized faces are quite disturbing when seen upright (see Fig. 2) but the disturbing effect is drastically reduced for inverted faces. This effect of disturbance-reduction due to inversion is less pronounced in participants with CP because (1) the effect for upright faces is not so striking for them in the first place and (2) they tend to notice the changes also in the inverted faces. In this BR version of the Thatcher effect, we expected that in in the control group the thatcherization of faces increases the saliency of faces and thus increases the dominance of the face stimuli. Inversion renders the Thatcherization effect much less obvious for normal observers and consequently should reduce saliency and dominance compared to the upright version. In contrast, in CP observers we expect a reduction in the inversion effects and thus also less of a difference between dominance values for upright versus inverted presentations. Thus, in sum we expected that the difference in the inversion effects expected between CG versus CP observers should be even more pronounced for thatcherized faces compared to normal faces (and thus we expected a significant three-way interaction). However, as will be seen below, our expectations were not met by the results.

**Figure 4:**
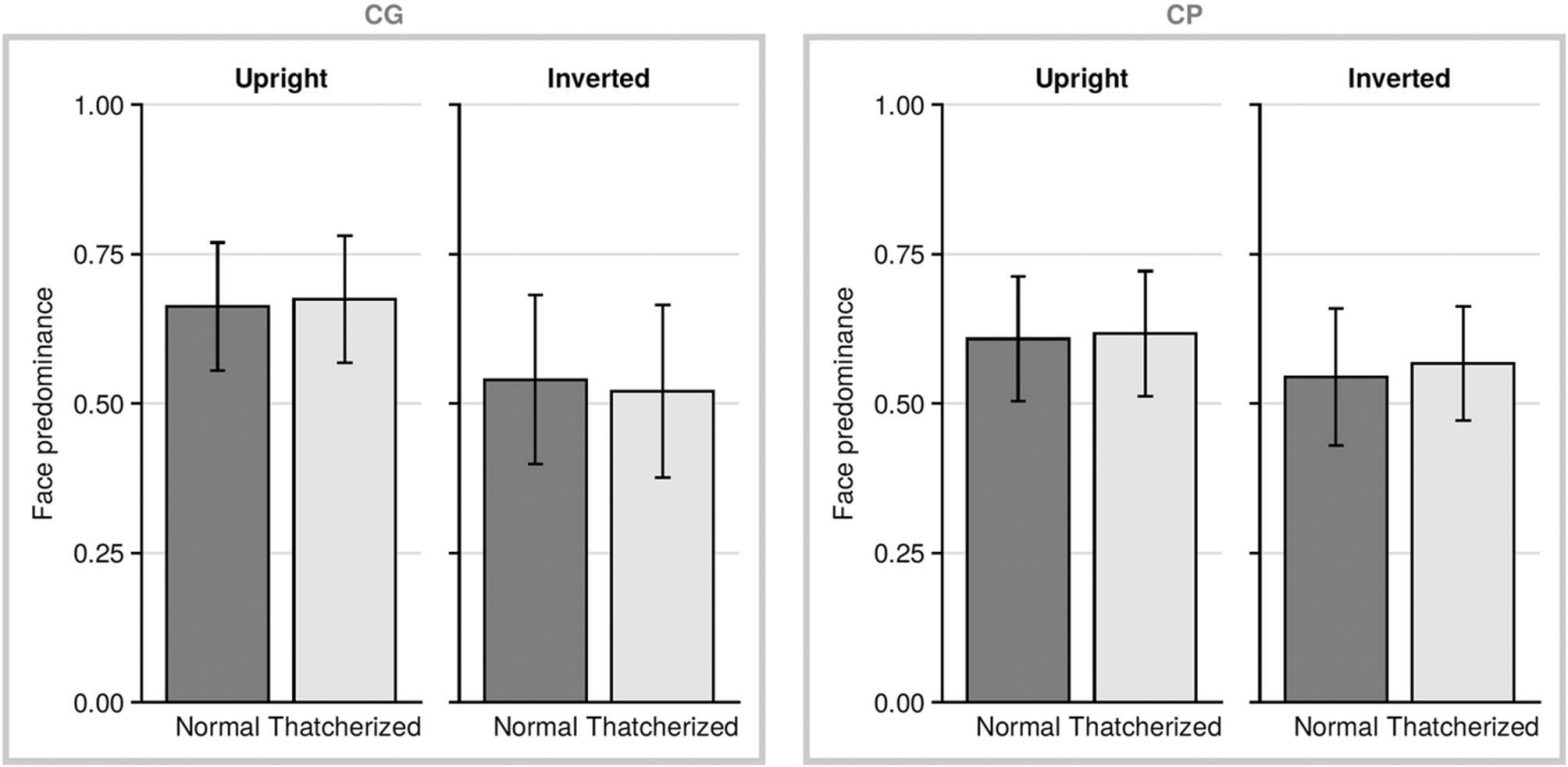
Face predominance in both groups (*CG* control group, *CP* congenital prosopagnosia) for normal faces (happy) and thatcherized faces in both inversions (upright, inverted).

Figure 4 shows no obvious effect of thatcherization but a clear effect of inversion which is in line with the assumption that CG observers rely on holistic processing strategy. Also, in accordance with the holistic processing strategy, the inversion effect in CP is less pronounced than in controls.

To investigate the differences between the two groups, we calculated a 2 × 2 × 2 ANOVA with the factors group (CP and CG), inversion (upright and inverted) and face-type (normal (happy) face or thatcherized face) using face predominance as the dependent variable. We only found a significant main effect of inversion (F(1,40) = 20.65, p < 0.0001, ηp^2^ = 0.34). The lack of a 3-way interaction indicates that our assumption about the saliency-enhancing effect of thatcherization was not warranted.

However, the marginally significant effect between group and inversion (F(1,1) = 3.6, p = 0.065, ηp^2^ = 0.082) suggests that the results are in accordance with our finding in Exp. 1; namely that face inversion shapes face predominance more in participants with normal face perception than in CP. The main effect of group was not significant.

We compared the inversion effect (inversion effect = face predominance upright faces—face predominance inverted faces) for both thatcherized faces (t(40) = 2.321, p = 0.019, *d* = 0.72) and non-thatcherized faces (happy faces) (t(40) = 1.240, p = 0.262, *d* = 0.38) between groups and found that only the inversion effect of thatcherized faces was significantly different between groups (i.e., higher in the control group than in the CP group, see Fig. 4). The finding for non-thatcherized faces is not surprising, as in Exp. 1 the inversion effect of happy faces also did not differ between groups (t(40) = 0.783, p = 0.914, *d* = 0.24)).

## Discussion

In the present study, we used BR to measure face processing in participants with congenital prosopagnosia and compared their perceptual dominance to unaffected observers. Our aim was to study face perception in CP implicitly to avoid the use of intentional strategies.

Generally, our results suggest that the face perception impairment in cp led to an overall decreased perception time for faces in BR. The lowered face predominance can be interpreted as reduced saliency of facial information due to the impaired face perception of this group. The inversion of faces, supposed to disturb holistic processing, affected controls in a more pronounced way than participants with CP, confirming this hypothesis using an implicit measure. Face predominance was affected by emotional facial expression in both groups, controls and CP. Upright fearful facial expressions increased dominance time compared to neutral faces. Concerning the question of whether inversion affected facial emotion processing differently in controls and CP, our results are indistinct, and we have to withhold judgment until further studies revisit this question.

In a second experiment, we intended to increase the saliency of faces by using the Thatcher effect, hypothesizing that this would lead to particularly pronounced differences between controls and participants with CP concerning the inversion effect. However, upright thatcherized faces did not further boost face saliency in our experimental setup and therefore we have currently no evidence to suggest that thatcherized faces provide a more sensitive test for the holistic face perception account as compared to the normal inversion effect.

However, the fact that we found clear inversion effect differences between CP and CG observers when using an implicit measure for perception, namely BR dominance, confirms that BR can be used to reveal visual deficits without having to rely on error measures of overt perceptual judgements. Such judgements and the corresponding errors might be influenced by strategies designed to either compensate for experienced deficits or to aggravate existing deficits (for example if testing takes place in the context of a legal dispute). BR could therefore be used as additional tool in the test arsenal of the visual neuropsychologist. The practical potential of BR is not necessarily undermined by the fact that we still do not know the exact mechanism underlying the abnormal dominance pattern in CP observers. It is possible that faces attract less attention for observers who find it harder to process them^34^. In recent years, BR has been applied to study perceptual alternations in groups with disorders such as neglect^88^, visual impairment^89^ or psychiatric disease^90^. To our knowledge, this is the first study that uses BR to investigate impairments in the field of visual agnosia and more particularly in prosopagnosia. Consequently, there is no data on CP and BR to compare our findings to, but there have been some approaches using implicit testing strategies on face perception that we will refer to when discussing the present results.

### Is CP an impairment of holistic face processing?

One account of CP assumes that the holistic process of face perception is selectively impaired. In this case one might expect to observe a reduced inversion effect on face dominance pattern in CP. Our results provide some support for this hypothesis. In both experiments we found that the inversion effect in CP observers was smaller than in CG observers. The scientific literature on this topic provides both evidence in support and evidence against the hypothesis that holistic processing is impaired in CP. Some studies report identical inversion effects in CP and controls^25,26^, other studies even suggest reversed effects, i.e., a preference or better performance for inverted faces in CP^21,23,24^. In both experiments of our study, CP observers differed from controls, but the effects were subtle, suggesting that holistic processing might not be lost but less effective, making observers with CP rely more on local features. It has been under debate whether holistic processing of faces is purely perceptual^31,91^ or has attentional components (e.g., Ref.^92^). As binocular rivalry is heavily influenced by attentional factors^93^, we cannot differentiate between these two positions based on our data. It is thus possible that our control group participants paid more attention to the upright faces compared to the inverted faces and that this difference in attention was larger than in our CP group.

### Facial emotion processing in CP

Another question that we wished to address relates to the emotion-decoding from facial expressions in CP observers. Our CP group showed no differences to the CG group in explicit emotion recognition in the Ekman 60 faces test. The reports of intact emotion-decoding of CP observers^10,49–51^ seem to stand in stark contrast with their difficulties in recognizing faces and are also unexpected given the established role of holistic processing strategies for the emotion-decoding of facial expressions as well as findings on impaired expression recognition in CP and in cases of acquired prosopagnosia^44–48,94–97^ One way to resolve this apparent conflict was provided in the last paragraph. (1) It is possible that holistic deficits in CP observers are not as pronounced and ubiquitous as previously assumed. Our own data provide partial support for this hypothesis: We find some indication for inversion effects in CP patients but also hints in the data suggesting that inversion and thus the contribution of holistic processes is reduced in comparison to what is found in CG.

A second possibility is that CP observers achieve emotion-decoding by different means than controls. (2) They might employ more explicit perceptual strategies and rely on less automatic means to decode emotions in faces. BR offers the possibility to test this in a task that does not explicitly require emotion decoding, i.e., the participants are not forced to extract which emotion is displayed because they merely report perceptual dominance. Thus, if emotion decoding is a more effortful task in CP, it might not happen if the task is implicit and does not require it. However, this prediction is not supported by our findings.

(3) A third option—i.e., that CP observers can decode emotion but rely more on local than holistic aspects—is also in line with our data. While the corresponding three-way-interaction in the main analysis failed to reach statistical significance (and thus limits the comparison to the CG), the fact that in CP the interaction between emotion and inversion was not significant (and the apparent similarity of the emotion effect in inverted and non-inverted faces found for CP but not CG, as shown in Fig. 3) suggest that the emotion-effect in CP observers might be less strongly affected by inversion.

If confirmed in a larger sample and supported by robust statistical findings such a result would suggest that CP observers can process emotions in faces automatically by relying either on still remaining holistic processes or by making more effective use of local features for emotion-decoding than controls. In case the latter interpretation was adopted one would have to accept the possibility that it is not just holistic but also local facial features that can be processed in an automatic fashion.

### The Thatcher effect

The Thatcher illusion is one of the most striking illusions of face perception. Observers are frequently unaware of the manipulation when presented with the faces in an inverted position and they are typically very surprised when presented with the same faces in an upright position. Given the robustness of those findings, we expected that thatcherization might have an even more pronounced impact on face dominance in BR than emotional expressions. However, this was not what we found. Thatcherization had no significant impact on performance in either control or CP observers. Given this failure it was not surprising that our other predictions relating to the Thatcher effect were also not borne out by our data. The most interesting question now is why such a subjectively highly salient effect leaves hardly any trace in the BR dominance pattern. One possibility is that our subjective impression is wrong: thatcherization might not be as attention-grabbing as we suspect. However, this assumption is undermined by the observation that attention-related ERPs are clearly modulated by this effect^98,99^. A second possibility is that BR is not a good measure of saliency. This is at odds with numerous studies showing that factors affecting saliency also affect dominance pattern in BR^63,65–68^. This leaves one other possibility: BR is specifically ill-suited to measure the specific saliency of thatcherization. Put differently, in the case of face dominance and thatcherization two opposing trends might cancel each other out. The one trend is that objects that are recognized as faces command more interest and attention than other objects. Thatcherized faces are particularly attention-grabbing, but this is partly achieved by virtue of those thatcherized faces appearing less like real faces. Another problem caused by the Thatcher effect itself, is that the thatcherized faces can only be designed from happy faces. We therefore had to use happy faces to investigate an effect caused by inversion, although previous results^68^ did demonstrate that happy faces do not show the most significant effect of inversion. The failure of thatcherization to have a significant impact on BR dominance pattern certainly deserves further research.

### Limitations

We should mention the limitations of our study. First, in phases of a change in dominant percept, the decision criteria of what is still a mixed and what is already an unambiguous percept (in this case face or house) in BR might differ from participant to participant even when identical instructions are given to all participants. Such variability in the adopted decision criterion might also cause intersubject variability in the predominance pattern. This is, however, a common problem in most BR paradigms. Second, the effect sizes were smaller than were anticipated, so some of the reported effects might suffer from a lack of statistical power given the present sample size. However, it should be noted that our sample size of 21 CP participants is large when compared to the majority of previous studies on CP (with sample sizes ranging somewhere between 1 and 10). Third, the differences in the relevant test scores for face perception for CP versus CG observers were statistically significant but still quite small. This can be explained by the fact that severity of CP varies widely between affected people and by the fact that some of the employed tests (example.g., the MFT) use test stimuli that show not just the faces but also provide additional visual information, such as hairstyle, that can be effectively used by CP observers to identify a person in spite of their prosopagnostic difficulties.

## Conclusion

BR is a useful implicit test to assess visual deficits in neurological participants. This technique might help to uncover intentional biases in perceptual performance and reveal deficits that are masked by increased effort or compensation in more explicit tests of perception. Our findings using BR to investigate face perception support the claim that CP comes with decreased but not total disruption of holistic face-processing. The findings also demonstrate that CP observers decode emotions despite their obvious problems in recognizing faces but cannot conclusively determine if this is based on global or local processing. Moreover, our results suggest that emotional decoding is an automatic process also in CP observers and thus our findings speak against the idea that this ability is based on the explicit use of a compensatory strategy, or that increased effort is needed. In short, our findings suggest that BR might prove a useful tool to uncover the subtle perceptual processing differences between typical observers and observers with congenital prosopagnosia.

### Supplementary Information

Supplementary Information.

## Data Availability

The datasets generated during and/or analysed during the current study are available in the OSF repository, [https://osf.io/e7z9v/].
