# Supplementary material for: Binocular rivalry reveals differential face processing in congenital prosopagnosia
Source: Sci Rep. 2024 Mar 20;14:6687. doi: 10.1038/s41598-024-55023-7 (PMC10954711; doi:10.1038/s41598-024-55023-7)
Supplement: Supplementary file 1 — Supplementary Information. [file 41598_2024_55023_MOESM1_ESM.docx]

**Supplementary Information:**


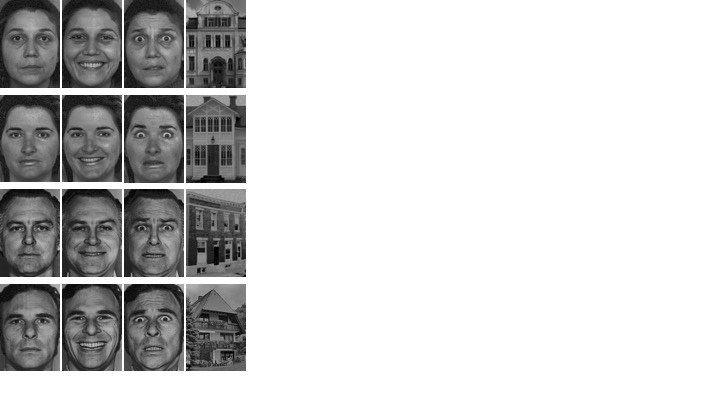


a) Stimulus material used in Exp. 1: two female and two male faces and the assigned houses were presented in upright and inverted orientation. All four identities were presented with different emotional expressions (neutral, happy, fearful).


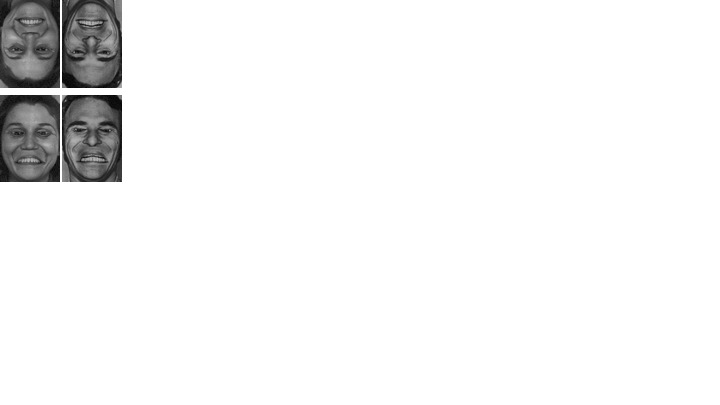


b) Stimulus material used in Exp. 2: thatcherized faces and normal faces were presented in upright and inverted orientations together with the assigned houses.
